# Semantic Knowledge Guided Diffusion Model for Biomedical Ontology Definition Generation

**DOI:** 10.1007/s41666-026-00240-0

**Published:** 2026-06-01

**Authors:** Shailesh Dahal, Ratri Mukherjee, Nicholas Mathews, Bijaya Adhikari, Kishlay Jha

**Affiliations:** University of Iowa, Iowa City, IA USA

**Keywords:** Diffusion model, Biomedical concept definition, Semantic knowledge

## Abstract

Biomedical ontologies such as the Unified Medical Language System (UMLS) play a crucial role in supporting practical applications such as predicting gene–disease associations, drug–drug interactions, and biomedical question answering. However, automatically maintaining and updating them remains a critical barrier as authoring high-quality textual definitions is both time-consuming and labor-intensive. As a result, UMLS ontology updates significantly lag behind the pace of scientific discovery, leaving many concepts without definitions or with outdated descriptions. Despite the pressing need for automated generation of UMLS concept definitions, this problem has largely remained unaddressed in prior research. To address this, we propose a novel diffusion-based approach for generating high-quality definitions of biomedical concepts. A key innovation in our approach is the adaptive semantic modulation mechanism, which dynamically adjusts the influence of semantic priors throughout the denoising process, enabling the model to flexibly balance local lexical fidelity with global semantic alignment. Extensive experiments on the largest available biomedical ontology dataset demonstrate that the proposed approach both significantly and consistently outperforms strong baseline algorithms across multiple evaluation metrics.

## Introduction

Biomedical ontologies such as the Unified Medical Language Systems (UMLS [1]) contain millions of concepts, including diseases, genes, drugs, and proteins, that play a crucial role in advancing scientific discoveries [2–4]. For example, studies have leveraged UMLS to find novel functional connections between genes, predict taxonomic expansion, and uncover adverse drug reactions [5, 6]. Despite the key role of UMLS in advancing biomedical research, maintaining and updating it remains a major challenge in the data mining community. This is primarily due to the continuous influx of novel biomedical concepts (e.g., emerging diseases, newly discovered genes, or experimental drugs) that require frequent updates to UMLS to accurately reflect the evolving landscape of biomedical knowledge [7]. A key step in updating UMLS is to efficiently generate definitions of thousands of newly curated biomedical concepts. However, at present, these definitions are manually written by domain experts at the National Institute of Health (NIH). This form of manual curation is time-consuming, expensive, and not scalable to large ontologies such as UMLS; yet, no existing works have attempted to generate the definition of biomedical concepts. To address this, we propose the novel task of automatically generating high-quality definitions of biomedical concepts present in ontologies such as UMLS.

Neural text generation [8] offers a plausible solution to the aforementioned task due to its excellent sequence modeling capacity to generate human-like text (e.g., concept definitions) given the input data (e.g., concept name). To this end, several autoregressive (AR) models [9] for text generation have been proposed for related tasks such as paraphrase generation [10], description generation [11], synonym generation [12], and data augmentation [13]. While AR models significantly improved the text generation quality, they still have certain drawbacks. First, AR models are prone to exposure bias, i.e., errors made early during the sequence generation can compound in later tokens. Second, AR models are not flexible for controlled text generation. This is limiting, especially for tasks such as biomedical text generation, where it is desirable to condition text generation on structured biomedical knowledge (e.g., semantic types [14]). To address these issues, more recently, diffusion models (DMs) [15], a class of generative models originally developed for high-quality image generation, have been introduced for non-autoregressive (NAR) text generation. In essence, DMs perform a multi-step denoising process to iteratively convert a random noise into a data sample. This iterative refinement allows DMs to incorporate complex control conditions more effectively than traditional AR models. This capability is particularly useful for current tasks where high-quality definitions must integrate both lexical information from concept names and structured biomedical knowledge, such as semantic types. By conditioning the denoising process on structured biomedical knowledge, DMs can dynamically balance lexical specificity and semantic abstraction across denoising timesteps. Moreover, recent advances in sampling acceleration [16] enable DMs to achieve a favorable trade-off between inference latency and generation quality, making them a promising framework for generating accurate, semantically-consistent biomedical definitions.

We propose a novel approach that leverages the structured semantic knowledge to guide the DM for generating high-quality concept definitions. Specifically, we propose to exploit the dual nature of semantic information available in biomedical ontologies: the lexical specificity provided by concept names and the semantic abstraction captured by ontological categories such as UMLS semantic types. These two forms of guidance offer complementary inductive signals. The first is grounded in the lexical surface form of biomedical concepts, such as the terminology used in a concept name. The second captures domain-level abstraction from structured ontological categories such as semantic types. Taken together, this strategy can significantly improve the fidelity, factual accuracy, and controllability of the generated concept definitions.

**Fig. 1:**
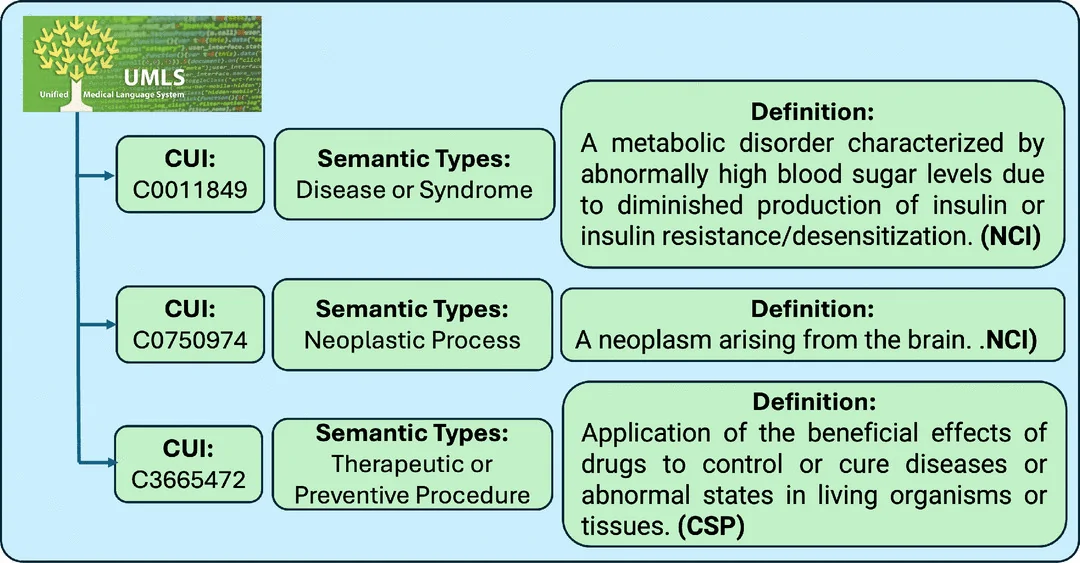
Examples of UMLS biomedical concepts showing their Concept Unique Identifiers (CUIs), associated semantic types, and expert-curated definitions from sources such as National Cancer Institute (NCI) and Computer Retrieval of Information on Scientific Projects (CRISP) Thesaurus (CSP)

As shown in Fig. 1, consider the concept *“C0011849”*, which corresponds to the term *“Diabetes Mellitus”* in UMLS. A high-quality definition should explicitly reflect its lexical identity (e.g., *“A metabolic disorder characterized by abnormally high blood sugar levels due to diminished production of insulin or insulin resistance/desensitization”*) while also implicitly aligning with its semantic type (e.g., categorized under *“Disease or Syndrome”*). Without access to both lexical and semantic knowledge, a model may generate fluent but factually incorrect or overly generic text.

To integrate both forms of knowledge effectively, our framework employs a *dual-guidance mechanism* that conditions the denoising trajectory on both concept names and semantic types. Specifically, we introduce an *adaptive modulation strategy* that dynamically adjusts the influence of semantic abstraction throughout the generation process, thereby emphasizing broad semantic structure at early diffusion steps and fine-grained lexical detail at later stages. This adaptive conditioning not only improves factuality and fluency but also enhances controllability and generalization to unseen biomedical entities. The proposed framework is intended to support biomedical research and ontology curation workflows, such as ontology completion and downstream clinical NLP applications, rather than direct clinical decision making.

Our key contributions can be summarized as follows.

- We make the first attempt to automatically generate concept definitions for biomedical ontologies such as UMLS. Specifically, we propose a novel diffusion language model that leverages semantic knowledge to guide the generation process and produce definitions that are both lexically precise and semantically meaningful.
- To address the varying relevance of semantic signals throughout the denoising trajectory, we propose a learnable adaptive gating function that adaptively modulates the strength of semantic-type conditioning. This unique strategy significantly enhances semantic alignment and fluency in the generated text.
- We conduct extensive experiments across standard biomedical benchmarks, demonstrating significant improvements over strong diffusion baselines.

## Related Works

Autoregressive (AR) LMs like GPT-2 [17], BART [18], and their domain-specific counterparts such as BioGPT [19], PubMedBERT [20] and BioBART [21] have demonstrated strong performance in biomedical generation tasks. While PubMedBERT specializes in biomedical masked language modeling, CBAG [22] introduces concept-based attention mechanisms tailored for biomedical definition generation, highlighting the importance of concept-grounded representations. These models generate tokens sequentially and excel in fluency, but often suffer from exposure bias and limited controllability.

DMs offer a fundamentally different paradigm for text generation by learning to reverse a noising process. This iterative refinement capability allows DMs to revisit and revise their outputs, offering potential solutions to exposure bias and error accumulation seen in AR generation. The primary challenge in adapting DMs to text is their inherent discreteness. Several lines of research have emerged to address this, broadly categorized into approaches that operate in continuous latent spaces and those that work directly on discrete tokens. Models like Diffusion-LM [23] introduced the first continuous DM in word embedding space, demonstrating controllability and training stability. DiffuSeq [24] adapts DMs for sequence-to-sequence (Seq2Seq) text generation by transforming discrete sentences into a continuous space. SeqDiffuSeq [25] improves this by implementing an encoder-decoder architecture, self-conditioning, and adaptive noise scheduling. LD4LG [26] and DiffLM [27] utilize encoder-decoder LMs to learn high-quality language autoencoders, enabling continuous diffusion in their latent spaces. PLANNER [28] combines latent semantic diffusion with AR generation to achieve global control over paragraphs by generating semantic paragraph embeddings in a coarse-to-fine manner within a latent space. These discrete DMs operate directly on discrete vocabulary tokens by performing denoising through masking or replacing tokens from a categorical distribution. RDM [29] offers a flexible framework with more effective training and decoding techniques by re-parameterizing the backward process. Diffusion-NAT [30] unifies pre-trained BART with discrete diffusion for a masked tokens recovering task, leveraging BART’s knowledge as a denoiser. More recently, hybrid approaches have emerged, adapting LMs to function as DMs. DiffuGPT and DiffuLLaMA [31], for instance, convert GPT2 and LLaMA [32] into DLMs through continual pre-training and attention mask annealing, leveraging their extensive linguistic knowledge. Despite their promise, these models typically rely on fixed conditioning, limiting their flexibility for domain-specific or multisource control, especially in biomedical applications, where hierarchical ontologies and concept labels offer rich, structured guidance. Controllable text generation has advanced from stylistic prompts to structured guidance using classifier-free diffusion mechanisms [33]. While multi-conditioning (e.g., TextSMOG [34]) has succeeded in vision and molecule domains, adapting such schemes to biomedical semantics remains limited.

## Methodology

**Fig. 2:**
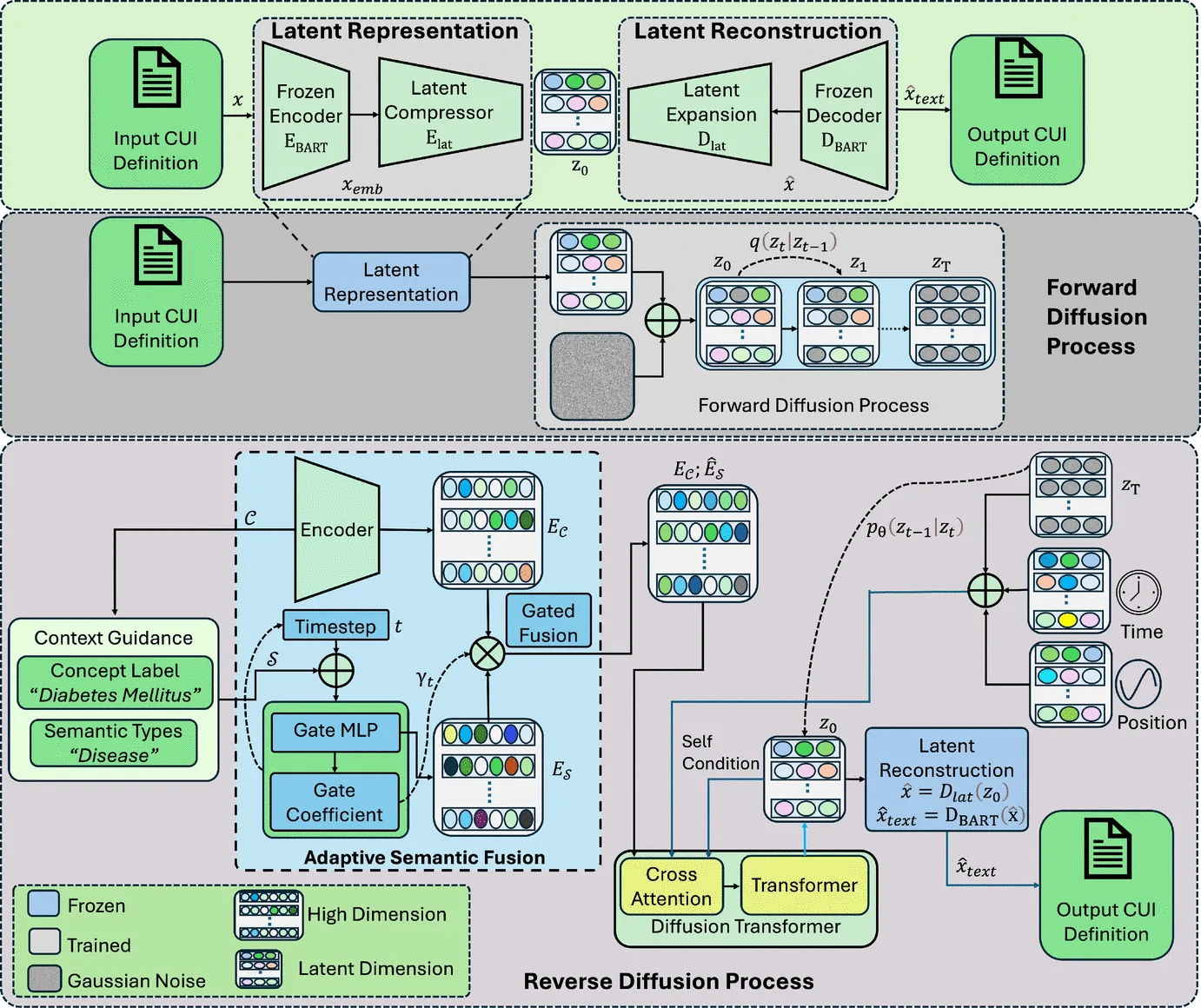
Overview of our semantic knowledge adaptive latent diffusion framework for biomedical ontology definition generation. The input biomedical ontology definition is encoded via a frozen BioBART encoder and compressed into a latent space. During training, Gaussian noise is added to the latent representation via a forward diffusion process. In the reverse process, a diffusion transformer denoises the latent using cross-attention over two structured knowledge sources: (1) concept-level guidance and (2) adaptively fused semantic knowledge embeddings. A learnable gating function modulates the influence of semantic knowledge over denoising steps. The final denoised latent is decoded to produce fluent biomedical definitions

Our goal is to develop a *Semantic Knowledge Adaptive Latent DM* designed to synthesize semantically coherent definitions for unseen CUIs. Our approach integrates structured biomedical knowledge into a latent denoising diffusion framework, enabling controlled and ontology-aligned text generation. Given a biomedical concept represented by its surface name and a set of associated semantic types, the task is to generate a free-text natural language definition that accurately describes the concept’s biomedical meaning. The model does not receive CUIs, synonyms, or gold definitions as input. During training, reference definitions serve only as supervision targets. At inference time, generation is conditioned solely on the concept name and optional semantic type information. Our objective is not to introduce new ontology or knowledge-representation formalisms, but to develop a generative learning framework that leverages existing ontological structure for controlled biomedical definition generation. We focus on UMLS because it provides a large collection of expert-annotated definitions suitable for training data-driven generative models. However, the proposed framework itself is not UMLS-specific and can be applied to other biomedical vocabularies by retraining on ontology-specific annotations, with performance depending on available data scale. An overview of the architecture is shown in Fig. 2.

### Preliminaries

Let $x \in \mathcal {X}$ denote a biomedical definition (e.g., a clinical statement or scientific description), composed of a variable-length sequence of tokens drawn from a vocabulary $\mathcal {V}$. We encode *x* as follows:

1. **Semantic Encoding via Pretrained BioBART.** We first encode *x* using a frozen biomedical encoder $E_{\text {BART}}$, such as BioBART, to obtain token-level contextual representations: $\textbf{x}_{\text {emb}} = E_{\text {BART}}(x) \in \mathbb {R}^{n \times d}$, where *n* is the number of tokens and *d* is the encoder’s hidden dimension. This step captures rich biomedical semantics, including lexical, syntactic, and contextual nuances, from large-scale biomedical corpora.
2. **Latent Compression via Autoencoder.** To map the high-dimensional contextual embeddings into a lower-dimensional latent space, we pass $\textbf{x}_{\text {emb}}$ through a latent encoder $E_{\text {lat}}$, based on a Perceiver architecture [35]. This yields a compact latent representation: $\textbf{z}_0 = E_{\text {lat}}(\textbf{x}_{\text {emb}}) \in \mathbb {R}^{k \times d_{\text {lat}}}$, where *k < n* and $d_{\text {lat}} < d$. The latent encoder is trained to preserve semantic fidelity while reducing redundancy. The resulting $\textbf{z}_0$ serves as the clean latent representation for the forward diffusion.

### Forward Diffusion in Latent Space

To model a generative prior over compact biomedical representations, we adopt the Denoising Diffusion Probabilistic Model (DDPM) [15] applied in latent space. This choice enables scalable generation while preserving semantic structure from upstream encoders. The forward diffusion process defines a fixed-length Markov chain that progressively corrupts a clean latent representation $\textbf{z}_0 \in \mathbb {R}^{k \times d_{\text {lat}}}$ by injecting Gaussian noise over *T* discrete timesteps. At each timestep $t \in \{1, \dots , T\}$, the noisy latent $\textbf{z}_t$ is sampled from a conditional Gaussian:

1 $$\begin{aligned} q(\textbf{z}_t \mid \textbf{z}_0) = \mathcal {N}(\textbf{z}_t; \sqrt{\bar{\alpha }_t} \, \textbf{z}_0, (1 - \bar{\alpha }_t) \, \textbf{I}), \end{aligned}$$

where $\{\alpha _t\}_{t=1}^T \in (0, 1)$ denotes a variance schedule, and $\bar{\alpha }_t = \prod _{s=1}^{t} \alpha _s$ is the cumulative product controlling the signal-to-noise ratio at timestep *t*. The forward process admits a closed-form reparameterization:

2 $$\begin{aligned} \textbf{z}_t = \sqrt{\bar{\alpha }_t} \, \textbf{z}_0 + \sqrt{1 - \bar{\alpha }_t} \, \boldsymbol{\epsilon }, \quad \boldsymbol{\epsilon } \sim \mathcal {N}(\textbf{0}, \textbf{I}). \end{aligned}$$

This forward process enables the model to learn denoising by simulating varying noise levels. To improve learning stability, we adopt a velocity-based parameterization [36], predicting an intermediate velocity $\textbf{v}$ that linearly combines signal and noise. During training, the timestep *t* is uniformly sampled and the model is trained to estimate $\textbf{v}$ from $\textbf{z}_t$. This denoising objective is further enhanced by our dual-guidance semantic conditioning framework, which injects structured biomedical knowledge as auxiliary context, detailed in Section 3.1.

### Reverse Diffusion and Generation

The generative model learns to reverse the forward process by estimating the velocity component at each timestep, conditioned on auxiliary information. The reverse denoising process is defined as:

3 $$\begin{aligned} p_{\theta }(\textbf{z}_{t-1} \mid \textbf{z}_t) = \mathcal {N}(\textbf{z}_{t-1}; \boldsymbol{\mu }_{\theta }(\textbf{z}_t, t), \boldsymbol{\Sigma }_{\theta }(\textbf{z}_t, t)), \end{aligned}$$

where $\boldsymbol{\mu }_{\theta }(\cdot )$ and $\boldsymbol{\Sigma }_{\theta }(\cdot )$ denote the predicted mean and variance at timestep *t*, parameterized by the model *θ*. Following prior work [36], we adopt a velocity prediction strategy, which improves training stability compared to direct noise prediction. The target velocity is defined as:

4 $$\begin{aligned} \textbf{v} = \sqrt{\bar{\alpha }_t} \cdot \boldsymbol{\epsilon } - \sqrt{1 - \bar{\alpha }_t} \cdot \textbf{z}_0, \end{aligned}$$

where $\boldsymbol{\epsilon } \sim \mathcal {N}(\textbf{0}, \textbf{I})$ is the Gaussian noise added during the forward process. The model is trained to minimize the mean squared error between the predicted and target velocity:

5 $$\begin{aligned} \mathcal {L}_v = \mathbb {E}_{\textbf{z}_0, \boldsymbol{\epsilon }, t} \left[ \left\| \textbf{v}_\theta (\textbf{z}_t, t) - \textbf{v} \right\| ^2 \right] . \end{aligned}$$

During inference, sampling begins from pure noise $\textbf{z}_T$, which is iteratively denoised using the learned velocity predictions to recover $\textbf{z}_0$. The final output is decoded in two stages:

6 $$\begin{aligned} \hat{x} = D_{\text {lat}}(\textbf{z}_0), \quad \hat{x}_{\text {text}} = D_{\text {BART}}(\hat{x}), \end{aligned}$$

where $D_{\text {lat}}$ is the latent-space decoder, and $D_{\text {BART}}$ reconstructs the final biomedical definition in natural language. This reverse process is conditioned on dual semantic signals: concept-level and adaptively modulated semantic-type embeddings introduced through cross-attention layers. This enables the model to generate lexically precise and semantically coherent definitions for previously unseen biomedical concepts.

### Dual Knowledge Conditioning and Adaptive Semantic Knowledge Guidance

Our approach is grounded in an adaptive and dual-conditioned latent diffusion framework that leverages both structured biomedical knowledge and temporal dynamics for controlled text generation. Specifically, we incorporate two complementary sources of semantic guidance: (1) *Concept Level Representations:* Lexical embeddings derived from the CUI surface form (e.g., “diabetes mellitus”) capture term-specific information, and (2) *Semantic Type Metadata:* Ontological labels (e.g., gene, disease, procedure) encode higher-order biomedical categories extracted from UMLS. Together, these signals guide the model to generate biomedical definitions that are both lexically precise and ontologically grounded. The conceptual embeddings act as anchors for target entities, while semantic types regularize generation with class-level structure. Rather than statically concatenating these signals, we introduce a principled mechanism to integrate semantic-type embeddings over time. Specifically, we define a learnable null prototype–a trainable embedding representing the absence of semantic type information. During training, we blend each real semantic type embedding with this null prototype using a time-dependent gating function, allowing the model to gradually shift its reliance on semantic abstraction throughout the diffusion process. Both conditioning streams are projected into a continuous space and injected into the reverse diffusion model via cross-attention, allowing nuanced and interpretable control while avoiding overconditioning.

#### Dual Conditioning

Let $\textbf{z}_t \in \mathbb {R}^{k \times d_{\text {lat}}}$ denote the noised latent at diffusion step *t*. We condition the model on two sources of structured knowledge:

1. **Conceptual Guidance** $\mathcal {C}$. The surface form of the target biomedical concept (e.g., *“diabetes mellitus”*) is encoded using a frozen BioBART encoder, yielding a contextual representation: $\textbf{E}_\mathcal {C} = E_{\text {BART}}(\text {CUI text}) \in \mathbb {R}^{k_c \times d},$ where $k_c$ is the token length of the concept name. This acts as a concept-specific anchor for cross-attention.
2. **Semantic Type Guidance** $\mathcal {S} = \{s_1, \dots , s_m\}$. Using MetaMap [37], we extract a set of semantic types associated with each concept. Each type $s_i \in \mathcal {S}$ is mapped to a trainable embedding: $\textbf{E}_\mathcal {S} = \{\textbf{e}_1, \dots , \textbf{e}_m\} \in \mathbb {R}^{m \times d},$ where each $\textbf{e}_i \in \mathbb {R}^d$ is a learned vector encoding class-level semantics (e.g., “Pharmacologic Substance”, “Disease or Syndrome”). These vectors serve as temporally modulated priors.

#### Adaptive Semantic Modulation

A core innovation of our model is the ability to modulate semantic-type conditioning dynamically over the denoising trajectory. Early in the diffusion process, the latent is heavily corrupted, and high-level semantic types offer useful structure. Later steps require finer lexical control. We capture this evolving need via a learnable adaptive gating mechanism.

Let $t \in \{1, \dots , T\}$ be the current diffusion step and define a normalized timestep, $\tau = \frac{t}{T} \in (0, 1)$. We compute a gating scalar $\gamma _t \in (0, 1)$ via a shallow feedforward network:

7 $$\begin{aligned} \gamma _t = \sigma \left( \textbf{W}_2 \cdot \textrm{SiLU}(\textbf{W}_1 \cdot \tau ) \right) , \end{aligned}$$

where $\textbf{W}_1 \in \mathbb {R}^{1 \times h}$, $\textbf{W}_2 \in \mathbb {R}^{h \times 1}$, and *σ* is the sigmoid activation. This gate determines the degree to which each semantic type should influence generation at step *t*. To implement this, we define a learnable null prototype $\textbf{e}_{\text {null}} \in \mathbb {R}^d$, which represents the absence of semantic-type guidance. Each real semantic embedding $\textbf{e}_i$ is adaptively interpolated as:

8 $$\begin{aligned} \tilde{\textbf{e}}_i(t) = \gamma _t \cdot \textbf{e}_i + (1 - \gamma _t) \cdot \textbf{e}_{\text {null}}, \quad \forall i \in \{1, \dots , m\}. \end{aligned}$$

This results in a modulated semantic-type embedding set:

9 $$\begin{aligned} \tilde{\textbf{E}}_\mathcal {S}(t) = \{ \tilde{\textbf{e}}_1(t), \dots , \tilde{\textbf{e}}_m(t) \} \in \mathbb {R}^{m \times d}. \end{aligned}$$

By softly controlling the contribution of high-level semantic categories, this mechanism allows the model to shift from semantic abstraction to lexical specificity over time, improving fluency, factuality, and controllability in generated definitions.

#### Latent Input and Self-Conditioning

Let $\textbf{z}_t \in \mathbb {R}^{k \times d_{\text {lat}}}$ denote the noisy latent at diffusion timestep *t*. In self-conditioning [38], the model is given an auxiliary input: its own estimate of the clean latent from a previous step, which helps stabilize training and improve temporal consistency. Specifically, we reuse the model’s estimate $\hat{\textbf{z}}_0^{t-1} \in \mathbb {R}^{k \times d_{\text {lat}}}$ from the previous timestep *t-1*, which was produced in the last iteration of the reverse process.

The current noisy latent $\textbf{z}_t$ and the self-conditioned estimate $\hat{\textbf{z}}_0^{t-1}$ are concatenated along the feature dimension to form the combined input: $\tilde{\textbf{z}}_t = [\textbf{z}_t \, \Vert \, \hat{\textbf{z}}_0^{t-1}] \in \mathbb {R}^{k \times 2d_{\text {lat}}}$ This combined latent is projected into the model dimension *d* through a learnable linear transformation:

10 $$\begin{aligned} \textbf{X}_t = \textbf{W}_{\text {proj}} \tilde{\textbf{z}}_t + \text {PosEmb}(k) + \text {TimeMLP}(t) \in \mathbb {R}^{k \times d}, \end{aligned}$$

where $\textbf{W}_{\text {proj}} \in \mathbb {R}^{2d_{\text {lat}} \times d}$ is a projection matrix, $\text {PosEmb}(\cdot )$ denotes absolute positional encodings, and $\text {TimeMLP}(\cdot )$ is a learnable MLP-based time embedding.

The resulting sequence $\textbf{X}_t$ encodes both current noisy information and a prior estimate of the clean latent, enriched with positional and temporal context. This hybrid representation allows the model to make smoother, temporally coherent denoising predictions as the reverse diffusion unfolds.

#### Knowledge Fusion

To integrate both lexical and ontological signals into the denoising process, we fuse the representations from Conceptual and Semantic guidance streams into a unified conditioning context. These embeddings are used as key-value pairs in the cross-attention layers of the diffusion transformer. Let $\textbf{E}_\mathcal {C} \in \mathbb {R}^{k_c \times d}$ be the contextual embedding of the CUI surface form (conceptual guidance), and $\tilde{\textbf{E}}_\mathcal {S} \in \mathbb {R}^{m \times d}$ be the time-modulated semantic type embeddings (semantic guidance). The fused context is constructed via concatenation:

11 $$\begin{aligned} \textbf{C} = \text {concat}(\textbf{E}_\mathcal {C}, \tilde{\textbf{E}}_\mathcal {S}) \in \mathbb {R}^{(k_c + m) \times d}. \end{aligned}$$

Corresponding attention masks $\textbf{M} = [\textbf{M}_\mathcal {C}; \textbf{M}_\mathcal {S}]$ are used to denote valid tokens within each context source. The projected latent sequence $\textbf{X}_t \in \mathbb {R}^{k \times d}$, defined in (10), is passed into the diffusion transformer as the query input. At each attention layer, cross-attention is applied between the latent queries and fused contextual keys/values:

12 $$\begin{aligned} \text {Attention}(\textbf{X}_t, \textbf{C}) = \text {Softmax}\left( \frac{\textbf{Q} \textbf{K}^\top }{\sqrt{d_h}} \right) \textbf{V}, \end{aligned}$$

where the projections are computed as: $\textbf{Q} = \textbf{X}_t \textbf{W}^Q , \quad \textbf{K} = \textbf{C} \textbf{W}^K , \quad \textbf{V} = \textbf{C} \textbf{W}^V$ with trainable projection matrices $\textbf{W}^Q, \textbf{W}^K, \textbf{W}^V \in \mathbb {R}^{d \times d_h}$. This cross-attention mechanism enables each latent token to selectively attend to relevant semantic signals–balancing lexical detail and categorical abstraction–guided by the denoising timestep. The resulting representations are both temporally grounded and semantically aligned, allowing the model to generate coherent and factually consistent biomedical definitions.

#### Probabilistic Interpretation of Dual Conditioning

We provide a probabilistic interpretation of our dual conditioning mechanism, which combines static conceptual guidance with adaptive semantic type guidance in a principled manner. This perspective frames the model’s reverse diffusion step as sampling from a Bayesian mixture of structured priors. Let $\textbf{z}_t \in \mathbb {R}^{k \times d_{\text {lat}}}$ denote the noisy latent variable at timestep *t*, and let $\mathcal {C}$ and $\mathcal {S}$ represent the conceptual and semantic-type conditioning signals, respectively. We interpret our dual-guidance mechanism as a mixture of structured priors, modulated over the diffusion trajectory. Specifically, we model the conditional generative process as:

13 $$\begin{aligned} p(\textbf{z}_t \mid \textbf{z}_0, \mathcal {C}, \mathcal {S}) \propto p(\textbf{z}_t \mid \textbf{z}_0, \mathcal {C})^{1 - \gamma _t} \cdot p(\textbf{z}_t \mid \textbf{z}_0, \mathcal {C}, \mathcal {S})^{\gamma _t}, \end{aligned}$$

where $p(\textbf{z}_t \mid \textbf{z}_0, \mathcal {C})$ is an *entity-specific prior*, grounded in the lexical identity of the target concept (e.g., “diabetes mellitus”), $p(\textbf{z}_t \mid \textbf{z}_0, \mathcal {C}, \mathcal {S})$ is a *semantically enriched prior*, incorporating both the concept name and its broader biomedical abstractions (e.g., “Disease”, “Pharmacologic Substance”), and $\gamma _t \in (0, 1)$ is the adaptive gating coefficient defined in (7), which modulates the degree of semantic abstraction over time. This leads to a log-linear interpolation in posterior space:

14 $$\begin{aligned} \begin{array}{c} \log p(\textbf{z}_t \mid \textbf{z}_0, \mathcal {C}, \mathcal {S}) = (1 - \gamma _t) \log p(\textbf{z}_t \mid \textbf{z}_0, \mathcal {C}) + \gamma _t \log p(\textbf{z}_t \mid \textbf{z}_0, \mathcal {C}, \mathcal {S}) + \text {const.} \end{array}\end{aligned}$$

This formulation captures two key properties: (1) The model always conditions on $\mathcal {C}$, anchoring generation around the specific biomedical entity, and (2) The influence of semantic types $\mathcal {S}$ is introduced softly and progressively via $\gamma _t$, allowing the model to transition from entity-specific detail to broader semantic generalization as denoising unfolds. In practice, we approximate these priors using the learned embeddings: $p(\textbf{z}_t \mid \textbf{z}_0, \mathcal {C})$ is instantiated by conditioning on the contextual embedding $\textbf{E}_\mathcal {C}$, and $p(\textbf{z}_t \mid \textbf{z}_0, \mathcal {C}, \mathcal {S})$ is instantiated by additionally incorporating the modulated semantic embedding $\tilde{\textbf{E}}_\mathcal {S}(t)$.

#### Training and Inference Strategy

We train the diffusion model to minimize the squared error between the ground-truth velocity and the predicted velocity from the transformer model, conditioned on both conceptual and semantic guidance:

15 $$\begin{aligned} \mathcal {L}_{\text {train}} = \mathbb {E}_{\textbf{z}_0, \boldsymbol{\epsilon }, t} \left[ \left\| \textbf{v} - \textbf{v}_\theta (\textbf{z}_t, t, \textbf{E}_{\mathcal {C}}, \tilde{\textbf{E}}_{\mathcal {S}}(t)) \right\| ^2 \right] , \end{aligned}$$

where $\textbf{v}$ is the target velocity as defined in (4), $\textbf{E}_{\mathcal {C}}$ is the concept-level embedding, and $\tilde{\textbf{E}}_{\mathcal {S}}(t)$ is the time-aware semantic-type embedding defined in (8). To improve robustness during inference, especially when some semantic signals may be missing, we apply classifier-free dropout during training. With a fixed probability, we randomly drop either $\textbf{E}_{\mathcal {C}} \rightarrow \emptyset$ (i.e., remove concept guidance), or $\tilde{\textbf{E}}_{\mathcal {S}}(t) \rightarrow \textbf{e}_{\text {null}}$ (i.e., remove semantic-type guidance), while optimizing the model with the same loss in (15). This allows the model to generalize across full, partial, or absent conditioning settings at test time. During generation, we apply classifier-free guidance (CFG) [33] to steer the model toward semantically consistent outputs. Specifically, we compute two velocity predictions, one under full conditioning and one unconditioned, and interpolate between them:

16 $$\begin{aligned} \textbf{v}_{\text {CFG}}(\textbf{z}_t, t) = (1 + \omega ) \cdot \textbf{v}_\theta (\textbf{z}_t, t, \mathcal {C}, \tilde{\textbf{E}}_{\mathcal {S}}(t)) - \omega \cdot \textbf{v}_\theta (\textbf{z}_t, t, \emptyset , \emptyset ), \end{aligned}$$

where $\omega \ge 0$ is a user-controlled guidance scale. In our experiments, we use *ω = 2* to achieve a favorable trade-off between fidelity and fluency. This guidance mechanism allows the model to flexibly control semantic adherence without sacrificing generation quality, further strengthening its ability to produce accurate, ontology-aligned definitions for biomedical concepts.

## Experiments

### Dataset

We choose our dataset from the 2025 release of the UMLS [1], focusing on the natural language definitions associated with CUIs. Each instance comprises a biomedical concept name, its expert-curated textual definition, and a set of associated semantic types extracted from MetaMap. This structured format allows the model to learn both fine-grained lexical meaning and higher-order semantic abstractions. The dataset consists of 277,956 samples, which we split into 222,364 training, 27,796 validation, and 27,796 test examples following an 80/10/10 ratio. We sample 1000 instances every 5000 steps from the validation set to assess the generation quality across multiple metrics.

### Evaluation Metrics

Specifically, we report BLEU [39], ROUGE–1/2/L [40], BERTScore (Precision, Recall and F1) [41] and Memorization. All metrics are computed using the standardized implementations provided by HuggingFace evaluate library to ensure consistency and reproducibility. $\text {ROUGE-N} = \frac{|\text {Matching N-grams}|}{|\text {Total N-grams in reference text}|}$ computes the recall-oriented overlap between generated and reference N-grams. We report ROUGE-1, ROUGE-2, and ROUGE-L, which respectively capture unigram, bigram, and longest common subsequence matches. BLEU measures N-gram precision with a brevity penalty (BP), focusing on fluency and surface similarity: $\text {BLEU} = BP \cdot \exp \left( \sum _{n=1}^{N} w_n \log P_n \right)$, $P_n$ is the N-gram precision. $\text {BERTScore(P, R, F1)}=\text {SIM}_{\text {BERT}}(x_{\text {gen}},x_{\text {ref}})$ evaluates semantic similarity by measuring the cosine distances in contextual BERT embedding space. Memorization Score measures the fraction of generated 4-grams that appear in the training set, serving as a tool for unintended data copying. The lower value indicates stronger generalization.

### Training Configuration

We train our model using a BioBART-based encoder-decoder backbone with 216M trainable parameters. The diffusion transformer employs a 12-layer architecture with 768-dimensional hidden states. We use a cosine learning rate schedule, warming up over the first 1000 steps. The model is optimized using AdamW with a learning rate of $2e^{-4}$, $\beta _1=0.9, \beta _2=0.999$, and weight decay of $1e^{-6}$. We enable self-conditioning with a *50%* probability and exponential moving average (EMA) updates. The training runs for 245K steps with a batch size of 128. We sample 1000 generations every 5000 steps and evaluate the performance. All experiments are conducted using a single NVIDIA A30 GPU (24 GiB).

### Baseline Algorithms

We compare our approach against four strong baseline algorithms. **Diffusion-LM** [23] applies continuous diffusion in embedding space by denoising Gaussian noise into word vectors. Although text is discrete, it leverages continuous representations to enable gradient-based control, but lacks domain-specific conditioning. **DiffuSeq** [24] applies diffusion over partially noised source token sequences while keeping the target intact. Both sequences are mapped to an embedding space, and a transformer-based model learns to reconstruct the clean source conditioned on the target. **SeqDiffuSeq** [25] improves sequence-to-sequence generation by adopting an encoder-decoder diffusion architecture. It integrates adaptive noise scheduling and self-conditioning to progressively refine the denoising process. **LD4LG** [26] models text generation in a compressed latent space using a latent autoencoder and a diffusion transformer.

## Results

### Main Results

**Table 1:** Main evaluation results on biomedical concept definition generation

Model	ROUGE *↑*			BLEU *↑*	BERTScore *↑*			PPL *↓*
	1	2	L		P	R	F	
Diffusion-LM	0.219	0.092	0.194	0.088	0.216	0.160	0.187	129.84
DiffuSeq	0.400	0.232	0.365	0.167	0.321	0.294	0.303	180.99
SeqDiffuSeq	0.494	0.335	0.466	0.309	0.400	0.360	0.380	148.75
LD4LG	0.517	0.349	0.483	0.295	0.462	0.427	0.444	144.36
Proposed Model	**0.549**	**0.390**	**0.515**	**0.333**	**0.487**	**0.455**	**0.471**	**115.47**

Bold indicates best performance

Metric abbreviations: *P* precision, *R* recall, *F* F1-score, *PPL* perplexity

Table 1 presents a comprehensive evaluation of our proposed dual-guided latent diffusion model against existing diffusion-based baselines across standard metrics, including ROUGE, BLEU, BERTScore, and perplexity. Our model consistently outperforms all baselines, achieving the highest scores on ROUGE-1 (0.549), ROUGE-2 (0.390), ROUGE-L (0.515), BLEU (0.333), and BERTScore-F1 (0.471), while also yielding the lowest perplexity (115.47). Among the baseline methods, LD4LG performs competitively, benefiting from its latent-space modeling, yet falls short across all metrics compared to our method. A consistent upward trend in generation quality is observed from the upper baselines to the lower ones, demonstrating progressive improvements in both lexical and semantic fidelity. While Diffusion-LM achieves the lowest perplexity (129.81) among the baselines, it significantly lags in all other metrics, indicating that low perplexity does not necessarily correlate with semantic correctness or informativeness. This suggests that Diffusion-LM may produce fluent text, but lacks domain-relevant content. DiffuSeq, which applies diffusion on discrete token embeddings for the seq2seq task, improves over Diffusion-LM across all generation metrics. However, its high perplexity and weaker semantic alignment limit its ability to generate coherent biomedical definitions. SeqDiffuSeq shows moderate performance due to its encoder-decoder design and self-conditioning, but lacks fine-grained control and ontology-aware conditioning. Diffusion-LM trails significantly in generation quality, especially in semantic-oriented metrics such as BERTScore and BLEU. Interestingly, it exhibits the lowest perplexity (129.84) among the baselines. This suggests that although Diffusion-LM can generate syntactically plausible token sequences, it lacks semantic richness and alignment with biomedical definitions, leading to underperformance in content-level evaluations. These results validate the effectiveness of our proposed framework, particularly the integration of dual guidance and adaptive semantic modulation in improving both lexical diversity and conceptual accuracy in biomedical definition generation.

### Analysis of Backbone Encoder-Decoder

**Table 2:** Reconstruction performance of compressed latent autoencoders using general BART vs. domain-specific BioBART

Enc. / Dec.	PPL *↓*		BLEU *↑*		R-1 *↑*		R-2 *↑*		R-L *↑*	
	AE	Ref	AE	Ref	AE	Ref	AE	Ref	AE	Ref
BART	48.318	46.255	0.874	0.937	0.955	0.972	0.917	0.957	0.954	0.972
BioBART	**46.351**	**45.977**	**0.896**	**0.954**	**0.961**	**0.982**	**0.928**	**0.968**	**0.960**	**0.982**

Abbreviations: *Enc. / Dec.* encoder / decoder, *PPL* perplexity, *R-1* rouge-1, *R-2* rouge-2, *R-L* rouge-L, *AE* autoencoder, *Ref* reference

To validate our choice of encoder-decoder backbone for the experiments, we compare standard BART and domain-adapted BioBART in an autoencoding setup, where both models reconstruct input definitions from compressed latent representations. As shown in Table 2, BioBART consistently outperforms general BART across all metrics, achieving lower perplexity (46.35 vs. 48.32), higher BLEU (0.896 vs. 0.874), and higher ROUGE-L (0.960 vs. 0.950). This reflects that BioBART’s domain-specific pretraining on biomedical corpora yields robust representations for specialized terminology under latent compression. While increasing latent dimensionality can further improve reconstruction fidelity, it substantially increases the computational cost of diffusion training and inference. We therefore adopt the *32× 64* configurations as a balanced operating point that preserves reconstruction quality while enabling efficient and stable diffusion modeling.

### Comparison with Autoregressive Models

**Table 3:** Comparison with autoregressive models on biomedical definition generation

Model	ROUGE *↑*			BLEU *↑*	PPL *↓*
	1	2	L		
BioGPT	0.161	0.061	0.126	0.036	60.3
PubMedBERT	0.507	0.363	0.478	0.169	214.9
GPT-4	0.287	0.102	0.227	0.057	27.7
GPT-5	0.299	0.103	0.231	0.055	38.4
LLaMA-3.1	0.285	0.095	0.228	0.038	**23.8**
Proposed Model	**0.549**	**0.390**	**0.515**	**0.333**	115.5

Bold indicates best performance

Abbreviations: *PPL* perplexity

We compare our proposed diffusion-based model with a broad set of AR baselines, including domain-specific models (BioGPT and PubMedBERT) and modern Large Language Models (LLMs) (GPT-4, GPT-5, and LLaMA-3.1), all evaluated under the same definition generation protocol. As shown in Table 3, general-purpose LLMs produce fluent and concise definitions with low perplexity (GPT-4: 27.7, GPT-5: 38.4, LLaMA-3.1: 23.8), but exhibit substantially lower lexical alignment with expert definitions (ROUGE-L *≈* 0.23, BLEU *≤* 0.057). BioGPT similarly attains low perplexity (60.3) but poor semantic overlap (ROUGE-L 0.126), while PubMedBERT achieves higher ROUGE-L (0.478) at the cost of very high perplexity (214.9), likely due to unstable decoding and limited controllability at generation time. In contrast, our model achieves the strongest overall balance, with the highest ROUGE-1/2/L (0.549 / 0.390 / 0.515) and BLEU (0.333), while maintaining moderate perplexity (115.5). These results highlight the importance of semantic grounding and controlled generation enabled by dual semantic guidance and adaptive modulation over surface-level fluency alone for ontology-aligned biomedical definition generation.

### Ablation Study

**Table 4:** Ablation study on the dual guidance mechanism in the proposed model

Model	ROUGE *↑*			BLEU *↑*	BERTScore *↑*			Mem *↓*
	1	2	L		P	R	F	
Proposed Model without Concept Guidance	0.209	0.084	0.186	0.092	0.038	0.032	0.035	0.534
Proposed Model without Semantic Type Guidance	0.496	0.339	0.466	0.284	0.444	0.407	0.425	0.485
Proposed Model	**0.549**	**0.390**	**0.515**	**0.333**	**0.487**	**0.455**	**0.471**	**0.463**

Metric abbreviations: *P* precision, *R* recall, *F* F1-score, *Mem* memorization

To quantify the impact of each knowledge source in our dual-guided latent diffusion framework, we perform an ablation study by selectively disabling either the concept-level guidance or the semantic type guidance. As shown in Table 4, removing conceptual guidance results in a drastic drop in generation quality, with ROUGE-1 dropping from 0.549 to 0.208 and BLEU from 0.333 to 0.091. This confirms that concept-level embeddings provide strong semantic anchors, which are especially crucial during early stages of denoising where the latent space is highly noisy. In contrast, ablating the semantic type guidance causes a less severe, still substantial, drop in performance. ROUGE-1 almost decreases by *10%*, and BERTScore-F1 score drops from 0.471 to 0.425. This validates our hypothesis that semantic types offer essential categorical priors that regularize the generation and improve factuality and coverage. Notably, the full model with dual guidance outperforms both ablated variants across ROUGE, BLEU, and BERTScore metrics, while simultaneously exhibiting lower memorization, showing strong performance and better generalization via structured, adaptive guidance.

Notably, even without semantic type guidance, the model maintains strong generation quality, indicating that semantic types function as auxiliary priors rather than required metadata. This makes the model robust to missing semantic information at inference time.

### Sensitivity Analysis

**Table 5:** Sensitivity analysis on semantic guidance strength (*γ*)

*γ*	ROUGE *↑*			BLEU *↑*	BERTScore *↑*		
	1	2	L		P	R	F
0.0	0.524	0.366	0.492	0.312	0.450	0.432	0.440
0.2	0.538	0.382	0.506	0.320	0.478	0.441	0.459
0.4	0.554	0.397	0.524	0.335	**0.500**	0.463	0.481
0.6	**0.556**	**0.398**	**0.525**	**0.337**	0.499	**0.464**	**0.481**
0.8	0.546	0.386	0.514	0.328	0.483	0.452	0.467
1.0	0.530	0.370	0.499	0.313	0.457	0.427	0.442

Metric abbreviations: *P* precision, *R* recall, *F* F1-score

To investigate the role of semantic guidance strength in our dual conditioning setup, we conduct a sensitivity analysis by manually fixing the semantic modulation scalar *(γ )* to values in $\{0.0,0.2, \dots , 1.0\}$ during inference. Notably, we perform this evaluation using a model checkpoint that was fully trained with the learnable time-dependent gating $\gamma _t$, but override it at inference with constant values to isolate its effect. As shown in Table 5, performance improves with increasing *γ*, peaking at around *γ =0.4-0.6*, and degrades slightly beyond this. This behavior aligns with our design intuition: low *γ* values underutilize semantic type guidance, while excessively high values might overweight noisy or irrelevant guidance. These results validate the utility of semantic modulation and dual knowledge guidance.

### Qualitative Evaluation

We conduct a qualitative inspection of the generated definitions to assess the model’s ability to produce accurate, semantically coherent, and contextually relevant descriptions across diverse biomedical concepts. Table 6 presents representative cases comparing reference definitions and model-generated outputs, conditioned on both concept name and semantic types. Our model-generated outputs align consistently with domain-specific terminology and definition structure. For instance, in “Decreased circulating fetuin A concentration”, the model accurately captures clinical semantics (“concentration below the lower limit of normal”) while preserving syntactic fluency. Similarly, for “Controlled Release Liquid Dosage Form”, the generated output accurately captures the necessary semantics like “pharmacological substance” through the phrase “release active and/or inert ingredients”. Even for abstract entities like “Endocrine”, the model successfully anchors the definition to physiological role, generating the phrase ”produce hormones”. These behaviors illustrate the effectiveness of semantic type conditioning and adaptive gating, which guide the model to generate accurate and semantically coherent biomedical definitions. Overall, the model demonstrates robust generalization to unseen concepts, often rephrasing or expanding beyond surface forms while preserving domain accuracy.

**Table 6:** Comparison of generated texts for UMLS dataset

Concept Name	Reference Definition	Semantic Types	Generated Definition
Decreased circulating fetuin A concentration	A reduction below normal of fetuin A in the blood circulation.	Daily or Recreational Activity, Finding, Manufactured Object	Concentration of fetuin A in the blood circulation below the lower limit of normal.
Controlled Release Liquid Dosage Form	A solution or suspension designed to release active and/or inert ingredient(s) at a controlled rate.	Natural Phenomenon or Process, Organic Chemical, Pharmacologic Substance	Liquid designed to release active and/or inert ingredient(s) so as to achieve a controlled release rate.
Endocrine	Collective designation for those tissues capable of secreting hormones.	Conceptual Entity, Hormone, Intellectual Product, Organization, Qualitative Concept, Tissue	Specialized organs or tissues that produce hormones.
Somatostatin Antagonist	Any agent that binds to and antagonizes somatostatin receptors.	Daily or Recreational Activity, Eukaryote, Finding, Functional Concept, Intellectual Product, Mental Process, Mental or Behavioral Dysfunction, Qualitative Concept, Quantitative Concept, Spatial Concept, Temporal Concept	Any agent that inhibits or blocks the activity of the proteolytic action of somatostatin.
Irrigators, Nasal/Nasal Sinus	Irrigators designed for automated or semiautomated irrigation and washing of tissue and/or debris from the nose and/or the nasal sinuses. These devices typically consist of a main electromechanical unit that includes a pump for irrigation/infusion.	Activity, Amino Acid, Biomedical Occupation or Discipline, Clinical Attribute, Conceptual Entity, Diagnostic Procedure, Disease or Syndrome, Educational Activity, Enzyme, Finding, Food, Functional Concept, Gene or Genome, Health Care Activity, Health Care Related Organization, Human-caused Phenomenon or Process, Idea or Concept, Immunologic Factor, Individual Behavior, Intellectual Product, Laboratory or Test Result, Manufactured Object, Medical Device, Occupational Activity, Pathologic Function, Patient or Disabled Group, Peptide, Plant, Professional or Occupational Group, Qualitative Concept, Quantitative Concept, Research Activity, Sign or Symptom, Temporal Concept, Therapeutic or Preventive Procedure, or Protein	Irrigators designed to deliver fluids into the nasal and/or nasal sinuses, providing access to the nasal cavity and nasal sinus during nasal surgery and/ori surgical procedures. These devices typically consist of a manual oscillator, a heater, and a system.

#### Qualitative Error Analysis

Table 6 illustrates both strengths and limitations of the proposed model. For concepts with well-defined biomedical semantics (e.g., *Decreased circulating fetuin A concentration, Controlled Release Liquid Dosage Form*), the generated definitions closely match the reference, indicating robustness to irrelevant semantic types. For abstract categories such as *Endocrine*, the model produces correct but relatively generic definitions, reflecting cases where semantic types are weakly descriptive (e.g., *Intellectual Product, Organization*) and provide limited guidance beyond the concept name. Two residual error patterns are observed. First, *Semantic Drift* occurs under extremely noisy semantic-type sets, as in *Somatostatin Antagonist*, where the definition deviates from receptor antagonism. Second, *Over and Under Specification* arises for complex terms (e.g., *Irrigators, Nasal/Nasal Sinus*), where additional plausible but unnecessary details are introduced. Overall, observed errors primarily stem from noisy or insufficient descriptive semantic type annotations rather than hallucination or grammatical failure. Semantic types extracted by MetaMap can be noisy or weakly descriptive, as illustrated in Table 6. While MetaMap is used in this study to obtain semantic types for UMLS concepts, the proposed framework itself is agnostic to the specific annotation tool and can leverage semantic category information from ontology-native metadata or alternative entity linking systems. In our framework, semantic types are treated as soft priors and incorporated via adaptive semantic modulation, a learnable null prototype, and classifier-free dropout, enabling the model to down-weight uninformative or conflicting types during denoising. As discussed above, such noise contributes to residual semantic drift and over- or under-specification, which are partially mitigated by adaptive conditioning but not fully eliminated. UMLS semantic types are defined at a fixed granularity by the Semantic Network (127 types). In our framework, each type is represented as a learnable embedding, which is effective at this level but may be less informative for ambiguous or noisy assignments.

#### Biomedical Correctness

Biomedical correctness in this study is evaluated relative to expert-curated ontology definitions. Generated definitions are compared against held-out UMLS definitions authored by biomedical experts, using both automatic semantic similarity metrics and manual qualitative inspection (Table 6). This evaluation assesses terminological alignment, semantic scope, and consistency with established biomedical knowledge. Under this evaluation, the generated definitions serve as semantically grounded draft descriptions aligned with expert-curated references, suitable for curator review while transparently characterizing their limitations.

## Conclusion

In this work, we propose a novel latent diffusion framework for generating high-quality textual definitions of biomedical concepts, addressing a critical gap in ontology completion for resources like UMLS. By leveraging dual guidance from concept names and semantic type labels, along with an adaptive semantic modulation mechanism, our approach enables controllable and semantically grounded text generation. Extensive evaluations show that our method consistently surpasses strong baselines across lexical and semantic metrics.

## Data Availability

The data used in this research are derived from publicly available biomedical ontology resources. Details of the data sources, preprocessing steps, and experimental settings are provided in the manuscript. To ensure reproducibility, the code and datasets are publicly available at: https://github.com/saileshdahal1/biongen.
